# Development of NULISA™ CNS Disease Panel 120 for comprehensive proteomic profiling of neurodegenerative diseases

**DOI:** 10.1002/alz.089847

**Published:** 2025-01-09

**Authors:** Xiao‐Jun Ma, Shweta Iyengar, Shalaka Deshmukh, Roopa Comandor, Sean Kim, Xiaomei Xu, Xioalei Qiu, Bingqing Zhang, Yuling Luo

**Affiliations:** ^1^ Alamar Biosciences, Fremont, CA USA

## Abstract

**Background:**

The development of reliable blood biomarkers for neurodegenerative diseases (NDDs) has been hindered by the lack of tools with sufficient sensitivity to detect low concentrations of brain‐derived proteins in plasma or serum in a highly multiplexed manner. NULISA™ (NUcleic acid‐Linked Immuno‐Sandwich Assay) has emerged as a promising solution, with attomolar sensitivity and capable of high multiplexing in a fully automated system. In this study, we introduce NULISA CNS Disease Panel 120, a 120‐plex NULISAseq assay for profiling key hallmarks of NDDs in both blood and cerebrospinal fluid (CSF).

**Methods:**

NULISA CNS Disease Panel 120 includes many established and emerging disease markers such as amyloid beta 38/40/42, phosphorylated Tau (p‐Tau181, p‐Tau217, and p‐Tau231), neurofilament light and alpha synuclein as well as APOE ε4 for APOE status. NULISA single‐plex assays for each target were developed and pooled into a 120‐plex assay with signal tuning to optimize detection of both low and high abundance targets simultaneously in both plasma and CSF samples. Performance assessment included target detectability, assay precision, and detection of differential abundance between disease and control groups using plasma (n=38) and CSF (n=29) samples from NDD patients and age‐matched controls.

**Results:**

We developed a CNS disease panel consisting of 120 targets linked to various pathways and processes characteristic of NDDs. Using just 10 µL of plasma or CSF, NULISA™ CNS Disease Panel 120 detected approximately 95% of targets in plasma and around 85% in CSF. It demonstrated high precision, with a median coefficient of variation (CV) of approximately 6.0%. Through linear regression analysis, we identified both known and novel proteins exhibiting significant differences in abundance between disease and age‐matched controls. Furthermore, the APOE ε4 assay accurately classified donor APOE status into carriers and non‐carriers using both sample types.

**Conclusion:**

We developed a comprehensive CNS disease‐targeted panel for the NULISA platform to meet the increasing demand for blood‐based biomarker discovery and validation. NULISA CNS Disease Panel 120 provides a powerful new research tool for the early detection and diagnosis of NDDs and developing novel therapeutic interventions.

